# 机器人支气管镜在周围型肺结节活检中的临床应用

**DOI:** 10.3779/j.issn.1009-3419.2024.106.08

**Published:** 2024-04-20

**Authors:** Baodong LIU

**Affiliations:** 100053 北京，首都医科大学宣武医院胸外科; Department of Thoracic Surgery, Xuanwu Hospital, Capital Medical University, Beijing 100053, China

**Keywords:** 周围型肺结节, 经支气管肺活检, 机器人, Peripheral pulmonary nodules, Transbronchial lung biopsy, Robot

## Abstract

随着胸部电子计算机断层扫描（computed tomography, CT）肺癌筛查的普及，周围型肺结节的检出率日益提高。部分患者通过获取活检标本明确诊断并及早治疗。经支气管肺活检是周围型肺结节非手术活检的方式之一，相较于经皮肺穿刺活检创伤小、并发症发生率低。然而，经支气管肺活检的诊断率约为70%，与经皮肺穿刺活检约90%的诊断率相比仍显逊色。2018年以来，机器人辅助支气管镜系统相继应用于临床，本文对其进一步提高周围型肺结节经支气管肺活检的诊断率加以综述。

## Abstract (en)

With the popularization of chest computed tomography (CT) lung cancer screening, the detection rate of peripheral pulmonary nodules is increasing day by day. Some patients could make clear diagnoses and receive early treatment by obtaining biopsy specimens. Transbronchial lung biopsy (TBLB) is one of the non-surgical biopsy methods for peripheral pulmonary nodules, which has less trauma and lower incidence of complications compared to percutaneous thoracic needle biopsy (PTNB). However, the diagnostic rate of TBLB is about 70%, which is still inferior to that of PTNB, which is about 90%. Since 2018, robot assisted bronchoscopy systems have been applied in clinical practice. This article reviews their application in further improving the diagnostic rate of peripheral pulmonary nodules by TBLB.

周围型肺结节的病理活检适应证参照Fleischner学会2017年肺结节管理指南和美国国立综合癌症网络（National Comprehensive Cancer Network, NCCN）肺癌筛查指南^[1,2]^。对直径≥15 mm的持续性纯磨玻璃结节（pure ground-glass nodule, pGGN）、直径≥8 mm的实性结节或实性成分≥5 mm的持续性部分实性结节（part-solid nodule, PSN），建议活检^[3]^。目前常用的非手术活检方法包括经皮肺穿刺活检（percutaneous needle biopsy, PTNB）和经支气管肺活检（transbronchial lung biopsy, TBLB）。

## 1 PTNB

Meta分析结果显示，PTNB诊断肺癌的敏感性为90%-92%，特异性为94%-99%^[4,5]^。回顾性研究^[6,7]^报告8家医疗机构的多中心结果，9384例初次PTNB（9239例患者）有27.6%（2590/9384）无法确诊，结节大小、实性成分、活检针的类型与之相关；并发现结节≤10 mm的PTNB诊断率为60.0%，亚实性结节（sub-solid nodule, SSN）的诊断率为64.4%，得出了PTNB对<10 mm的SSN不太有效的结论。尽管PTNB的诊断率高，但是并非没有风险^[8,9]^，并发症包括气胸、出血、针道种植等。气胸最常见，发生率为21.7%-38.4%，其中1.5%-3.0%的患者需要放置胸腔闭式引流管。出血占第2位，包括肺内出血、咯血和血胸等，其中咯血发生率为5.2%-11.1%，有17.8%（11.8%-23.8%）的患者需要输血；血胸发生率为0.57%-1.5%。针道种植非常罕见。空气栓塞发生率为0.02%，死亡率高，致残率高。因此，TBLB逐渐得到重视。

## 2 TBLB

TBLB作为PTNB技术无法病理诊断持续性周围型肺结节的补充，越来越受到重视。引导包括手绘、X线透视、锥形束电子计算机断层扫描（cone beam computed tomography, CBCT）、超细支气管镜、虚拟支气管镜导航（virtual bronchoscopy navigation, VBN）[包括电磁导航支气管镜（electromagnetic navigation bronchoscopy, ENB）]、机器人辅助支气管镜（robotic-assisted bronchoscopy, RAB）等到达病变（to the lesion）；确认工具到达病变（tool-in-lesion）包括径向支气管内超声探头（radial endobronchial ultrasound, r-EBUS）、经支气管超声导向鞘引导（endobronchial ultrasound-guide sheath, EBUS-GS）、X线透视、C型臂断层融合、CBCT和ENB，降低CT-人体偏差（CT-to-body divergence, CTBD），联合现场快速细胞学评估（rapid onsite cytologic evaluation, ROSE）技术，对位于支气管分叉没有支气管征者的结节采用支气管镜下经肺实质结节隧道（bronchoscopic transparenchymal nodule access, BTNA）技术；取材方法包括活检钳、穿刺针、细胞刷和冷冻活检等，前两者真阳性率最高（86.9%和86.6%）^[10]^。

### 2.1 ENB

2018年Folch等^[11]^进行的前瞻性多中心队列NAVIGATE研究纳入了29家医院的1215例患者，评价X线透视或EBUS引导下ENB诊断肺结节的效率，其中49.1%的患者结节直径<20 mm，ENB辅助获取的标本中44%为恶性，诊断肺癌的敏感性为69%，特异性为100%，阳性预测值为100%、阴性预测值为56%；1年后确认的诊断准确率为73%，气胸的发生率为4.3%，出血的发生率为2.5%。NAVIGATE研究^[12]^的2年随访结果提示诊断率较前降低，约为67.8%（61.9%-70.7%，欧洲55.2%，美国69.8%），恶性敏感性为 62.6%，I至II期肺癌占64.7%（欧洲55.3%，美国65.8%）。气胸和支气管肺出血的发生率分别为4.7%和2.7%（3.2%和1.7%需要干预或住院治疗），呼吸衰竭发生率为0.6%。

一项纳入17项研究共1106例患者的meta分析^[13]^发现，ENB的合并诊断敏感性、特异性、阳性似然比、阴性似然比和诊断比值比（odds ratio, OR）分别为82%、100%、19.36、0.23和97.62；总受试者工作特征曲线（summary receiver operating characteristic curve, SROC）的曲线下面积（area under the curve, AUC）为0.9786；未发现操作相关并发症。另一项纳入40项研究^[14]^共3342例患者的meta分析发现，ENB在90.9%（95%CI: 84.8%-96.9%）的辅助测试中获得了足够的样本，诊断恶性肿瘤的合并敏感性为77%（95%CI: 72%-82%）和特异性为100%（95%CI: 99%-100%）；SROC显示AUC为0.955（P=0.03）；发生气胸的风险为2.0%（95%CI: 1.0%-3.0%）。

### 2.2 EBUS-GS

2004年Kurimoto等^[15]^的前瞻性开放研究中，入组150个周围型肺结节，评价EBUS-GS引导下诊断肺结节的效率，77%的患者诊断为恶性，穿刺针位于结节内和结节外诊断肺癌的敏感性分别为87%和42%，中度出血的发生率为1%。

### 2.3 r-EBUS

在一项采用r-EBUS引导下诊断周围型肺结节的单中心回顾性研究中^[16]^，2008至2019年2735例患者中的1627例最终被诊断为肺癌，r-EBUS引导比例从2008年的25%上升到2019年的92%，支气管征从2009年的100%下降到2019年的80%（P=0.001），总诊断率为74%，有支气管征时为80%。

一项纳入51项研究共7601例患者的meta分析^[17]^中，r-EBUS合并敏感性为0.72（95%CI: 0.70-0.75），SROC的AUC为0.96（95%CI: 0.94-0.97），观察到显著的异质性（I^2^=76%，异质性P<0.01）；未发现敏感性与支气管征、结节大小、X线透视、VBN、导向鞘、癌症患病率的关联，但是ROSE与敏感性增加有关（P=0.01）；气胸发生率为0.7%（95%CI: 0.3%-1.1%）。

## 3 TBLB和PTNB比较

一项回顾性队列研究^[18]^分析2013至2015年149例PTNB和146例ENB的诊断率和并发症发生率，结果前者较后者的诊断率高（分别为86.0%和66.0%，P<0.001），前者操作时间短（P<0.001），两组的主要并发症发生率相似（症状性出血，需要放置胸腔闭式引流管和/或需要住院的气胸）。另一项回顾性队列研究^[19]^经过倾向匹配评分发现二者诊断率没有统计学差异（P=0.124），但PTNB的并发症发生率显著高于ENB（P<0.001）。

两项系统性回顾评估r-EBUS和PTNB对周围型肺结节的诊断率^[20,21]^，r-EBUS的合并诊断敏感性为69%-75%，低于PTNB的93%-94%；对≤20 mm的周围型肺结节，PTNB的合并诊断率为92%，优于r-EBUS的66%；对>20 mm但≤30 mm的周围型肺结节，r-EBUS的合并诊断率提高到81%；r-EBUS的手术相关并发症发生风险较低。

2012年meta分析^[22]^中共纳入39项研究中的3052个结节。研究ENB、VBN、r-EBUS、超细支气管镜和/或r-EBUS-GS对周围型结节的诊断率；合并诊断率为70%，效率随着结节大小增加而增加；气胸发生率为1.5%；引导支气管镜技术的诊断率仍然低于PTNB。2023年meta分析^[23]^中纳入126项研究16,389个结节，2012年前39项研究3052个结节（诊断率70.5%），2012年后87项研究13,535个结节（诊断率69.2%），诊断率无统计学差异（P>0.05）；结节>20 mm、存在支气管征和恶性肿瘤高患病率与诊断率高有关。

总之，TBLB总体诊断灵敏度明显低于PTNB（~70% vs 90%左右）；气胸发生率PTNB约为20%，TBLB约为3%。r-EBUS和ENB的诊断率不能令人满意^[24,25]^，其原因可能如下：首先超细支气管镜联合EBUS-GS的i-LOCATE研究中，其超声外观显示肺不张与目标结节相似；其次，CT采集在吸气末和机械通气功能残气量的肺容积存在差异；再次，操作条件需要较高的PEEP、较低的吸入氧浓度（fraction of inspiration O_2_, FiO_2_）和6-8 mL/kg的潮气量，潮气量呼吸时，接受导航支气管镜检查患者的结节在吸气和呼气条件下两次扫描之间平均偏移为17.6 mm^[25]^。当偏差定义为CT采集目标位置与实时3D影像目标结节位置重叠<10%时，50%的结节存在偏移；当偏差定义为虚拟目标位置与实时目标位置相差10 mm时，60%的结节存在偏移^[26]^。为减少CTBD，提高周围型肺结节TBLB的诊断率，机器人辅助支气管镜（robotic-assisted bronchoscopy, RAB）应运而生。

## 4 RAB

2018年，Auris Health推出的Monarch^®^系统获得美国食品药品监督管理局（Food and Drug Administration, FDA）认证；2019年，Intuitive Surgical公司研发的Ion^®^系统获得FDA批准；2023年，Noah Medical公司研发的Galaxy^®^系统获得FDA批准。

### 4.1 Monarch^®^系统

有2个独立的机器人手臂，可以同时或单独操作；直径4.2 mm的支气管镜插入直径6 mm可130°活动的鞘管，并从鞘管中伸出并实现180°弯曲，实现4个方向运动；支气管镜依靠磁导航及气管镜镜头的微型摄像机引导进入气管，实现操作的可视化及定位；在导航至目标肺结节后可以将鞘管锁定在适当的位置；支气管镜内含有直径2.1 mm工作通道，可实现r-EBUS、活检、刷检、吸引等操作。

在2具人尸体比较相同外径传统细支气管镜和Monarch®系统的REACH尸体验证研究^[27]^中，通过电磁导航和透视测量支气管生成计数和插入深度，Monarch®系统在所有肺段都优于传统细支气管镜，尤其是在角度增加的支气管，如右肺尖段支气管（平均生成计数分别为8和3.5）和左肺尖后段支气管（平均生成数为8和4.5）。

在植入67个直径10-30 mm（平均20.4 mm）人造肿瘤的8具人尸体上进行的首个关于准确性的ACCESS研究^[28]^中，术前进行了CT扫描，所有尸体都进行了气管插管和机械通气；手术由一名支气管镜医生对每具尸体进行Monarch®系统下ENB、r-EBUS和X线透视检查，以定位和活检周围型肺结节；总体诊断率为65/67（97%），与21-30 mm的结节、同心或偏心的r-EBUS图像或与胸膜的相对距离相比，<20 mm的结节的诊断率没有统计学差异。

首个关于Monarch®系统安全性可行性的前瞻性单中心Feasibility队列研究^[29]^结果显示，研究者的中位年龄为67岁（38-79岁），15例肺结节患者进行活检（12例周围型病变，3例中央型病变），没有发生严重急性呼吸系统综合征，包括气胸和严重出血，活检样本取自93%的患者；60%（9/15）的患者确诊为癌症；操作时间前5例为45 min（21-84 min），后9例为20 min（7-47 min）（P=0.039）。

首个大型多中心回顾性研究^[30]^分析Monarch®系统联合X线透视对165例患者的167个肺结节导航活检情况显示，70.7%的病变位于肺外周1/3，支气管征为64%；88.6%的病变成功导航（r-EBUS），组织样本获取率为98.8%，r-EBUS图像位于中心、偏心和缺失的效率分别为81.5%、71.7%和26.9%，诊断率为69%-77%；术后6例（3.6%）发生气胸，4例（2.4%）需要放置胸腔引流管，4 例（2.4%）活检后出血；没有呼吸衰竭、死亡或其他任何与手术相关的并发症发生。

在一项包含264例患者的回顾性研究^[31]^中，结节中位大小为19.3 mm，58.9%位于外周，30.1%有支气管征；99.6%的患者通过Monarch®系统获得样本，病理学诊断恶性肿瘤为115例（43.6%），良性为110例（41.7%），未诊断为39例（14.8%）；指数诊断率为85.2%（95%CI: 80.9%-89.5%），12个月诊断率为79.4%（95%CI: 74.4%-84.3%）；替代定义指数诊断率估计值为58.7%（95%CI: 52.8%-64.7%），12个月诊断率89.0%（95%CI: 85.1%-92.8%）；恶性肿瘤敏感性为79.3%（95%CI: 72.7%-85.9%），12个月后癌症患病率为58.0%。

在一项包括124例患者的回顾性研究^[32]^中，结节中位大小为20.5 mm，75%有支气管征；所有患者至少随访12个月，总体诊断准确率为77%（95/124）；同心、偏心和缺失的r-EBUS图像的诊断准确率分别为85%、84%和38%（P<0.001）。在多变量分析中，r-EBUS阳性图像和结节大小为20-30 mm的患者获得诊断的概率更高；12个月恶性肿瘤的诊断准确率、敏感性、特异性以及阳性和阴性预测值分别为77%、69%、100%、100%和58%；2例（1.6%）患者出现气胸，4例（3.2%）患者出血；未发现术后呼吸衰竭。

首个前瞻多中心可行性BENEFIT研究报道5个中心采用Monarch^®^系统联合X线透视诊断55例周围型肺结节研究^[33]^结果显示，其中1例患者撤回知情同意，仅54例患者纳入分析，结节中位大小为23 mm，59%有支气管征；53例患者可获得r-EBUS图像，其中51例（51/53, 96.2%）成功导航，诊断率为74.1%；术后2例（2/54, 3.7%）发生气胸，1例（1.9%）需行胸腔闭式引流。

### 4.2 Ion^®^系统

属于单个机械臂控制的支气管镜，鞘管外径 3.5 mm，工作通道外径2.0 mm；镜身头端植入视觉探头，可实现180°弯曲镜身到达肺的所有18个段，形状传感器的实时测量与机器人控制算法相结合，在导航至目标结节后，可以将鞘管锁定在适当的位置；但在组织采样之前必须移除探头，无法实现操作的实时反馈；可使用r-EBUS、活检针、活检钳、毛刷活检；可以记录活检针的活动轨迹，方便实现多次活检，提高活检准确度。

在植入20个直径<20 mm人造肿瘤的5具人尸体验证的前瞻性单盲随机对照PRECISION-1研究^[34]^中，6名医生进行60次操作，用超细支气管镜联合r-EBUS，对比ENB和Ion^®^系统对目标结节进行定位和穿刺的能力，与ENB相比，使用Ion^®^系统的定位和穿刺成功率更高（80% vs 45%, P=0.02）；在穿刺失败的病例中，三组之间穿刺针到目标结节的误差距离差异有统计学意义（P=0.0014）。

在人尸体准确性验证的前瞻性单盲临床前ACCURACY研究^[35]^中，结节中位大小为16.2 mm，16个结节共行38次RAB手术；所有目标结节位于肺外1/3处，31.3%有支气管征；当Ion^®^系统鞘管尖端更靠近结节时，中心靶点命中率提高（<10 mm为68%，10-20 mm为66%，20-30 mm为11%，P<0.001）；多变量分析证实，中心靶点命中率的最强预测因素是Ion^®^系统鞘管与结节的距离（每增加1 mm，OR=0.89，P<0.001），与支气管征、分叉或同心r-EBUS图像的存在无关。

Fielding等^[36]^报道了首个Ion^®^系统的安全性可行性研究，纳入29例患者，结节平均大小为12 mm，无支气管征者占41.4%；其中28例（96.6%）成功导航；随访时间为6个月，总体诊断率为79.3%，恶性肿瘤的诊断率为88%；没有发生过气胸或在手术过程中观察到严重出血。

在一项包含112例患者120个结节的回顾性研究^[37]^中，针吸活检、活检钳和冷冻探针诊断率为90%；近18%的诊断完全来自冷冻活检样本，优于针吸活检和活检钳。在一项包含241例患者270个结节的前瞻性多中心试验^[38]^中，结节最大平均直径为（18.8±6.5）mm，平均注册和导航时间从前10例的10 min减少到随后的7 min；8例（3.3%）发生无症状气胸，1例（0.4%）放置引流管；2例（0.8%）患者出现气道出血，均在填塞后5 min内消失。

在一项包含60例患者65个结节的多中心单臂前瞻性PRECIsE研究^[39]^中，操作时间为66.5 min[四分位距（interquartile range, IQR）：50.00，85.5]，从鞘管尖端到虚拟结节最近边缘的距离为7.0 mm（IQR: 2.0, 12.0），活检完成率为97.0%，未报告任何级别的气胸或气道出血。

### 4.3 Galaxy^®^系统

内置摄像头的一次性使用支气管镜由单个机械臂控制的单个支气管镜组成，能够与C臂或锥形束CT无缝衔接；鞘管外径4 mm，工作通道2.1 mm，可实现镜身180°弯曲，使其可以到达肺的所有18个段；支气管镜在磁导航引导下到目标结节2 cm以内，然后通过标准荧光C型臂使用专有的CTBD校正、透视增强、Tool-in-lesion功能（TiLT+技术）来确认病变，可实现r-EBUS、活检、刷检、吸引等操作。目前公开发表的论文较少^[40]^。

### 4.4 影像辅助RAB

见表1^[41⇓⇓⇓-45]^。

**表 1:** 影像辅助机器人气管镜的研究

Author	Platforms	Technology	Sample size	Diagnostic yield	Malignant sensitivity	Accuracy	Adverse events
Reisenauer et al.^[41]^	Ion^®^ robotic platform with CIOS	Robotic platform for navigation with 3D multiplanar fluoroscopy for intra-procedural real-time guidance	30	93.3%	73.3%	-	-
Kalchiem-Dekel et al.^[42]^	Ion^®^ robotic platform with CIOS	Robotic platform for navigation with 3D multiplanar fluoroscopy for intra-procedural real-time guidance	159	81.7% (130/159)	83.3%	-	3.0% Pneumothorax: 1.5%
Benn et al.^[43]^	Ion^®^ robotic platform with CBCT	Robotic platform for navigation with intra-operative CBCT for biopsy tool guidance	59	83% (49/59)	84% (31/37)	86% (51/59)	Pneumothorax: 3.8%
Styrvoky et al.^[44]^	Ion^®^ robotic platform with CBCT	Robotic platform for navigation with intra-operative CBCT for biopsy tool guidance	209	-	-	91%	Pneumothorax: 1% Drainage: 0.5%
Cumbo-Nacheli et al.^[45]^	Monarch^®^ robotic platform with CBCT	Robotic platform for navigation with intra-operative CBCT for biopsy tool guidance	20	-	86.6% (13/15)	-	-

CBCT: cone-beam computed tomography; 3D: 3-dimensionsal.

### 4.5 RAB诊断率

RAB对周围型肺结节的导航成功率为88%-96%，50%没有支气管征，诊断率为69%-86%，针吸为100%，钳检为70%-96%；其中Monarch®系统诊断率为70%左右，而Ion^®^系统超过了80%；ROSE的应用，使得很大一部分肺结节得以现场诊断，并减少了其他设备的应用^[46]^。在一项纳入19项队列研究，共1542例患者1697个肺结节的meta分析^[47]^中，13项研究使用Ion^®^系统，6项研究使用Monarch^®^系统，目标肺结节的平均最大径为16.22 mm（95%CI: 10.98-21.47），平均操作时间为61.86 min（95%CI: 46.18-77.54）；总体诊断率为84.96%（95%CI: 62.00-95.00），恶性敏感性为81.79%（95%CI: 43.00-96.00）；并发症发生率为4.76%（95%CI: 2.00-15.00）；两种系统间各结局指标差异无统计学意义。

一项多中心回顾性研究^[48]^比较了RAB肺活检和PTNB的临床结果和并发症，共有225例患者入组（RAB肺活检为113例，PTNB为112例）；二者总诊断率分别为87.6%和88.4%；RAB对恶性疾病肺活检的敏感性为82.1%，特异度为100%；PTNB的敏感性为88.5%，特异性为100%；PTNB的并发症发生率明显高于RAB肺活检（17% vs 4.4%, P=0.002）。

### 4.6 RAB安全性

在安全性方面，部分研究报告无术后并发症，而部分研究报告有1.5%-3.8%的气胸发生率，气道出血发生率更低（0.8%-2.4%），显示了RAB的高度安全性，与其他引导支气管镜技术无明显差异。

## 5 RAB优势和局限性

Akulian等^[49]^开展ATLAS研究对比Ion^®^系统和ENB不同导航规划平台的差异性，结果表明Ion^®^系统导航通路的末端到目标周围型肺结节的距离最小（9.4 vs 14.2 vs 17.2 mm）；提示RAB较ENB具有更高的靶结节定位、活检成功率；ENB的平均手术时间短于30 min，RAB平均操作时间为60 min。因此针对周围型肺结节取样活检，RAB相对于ENB及传统支气管镜，具有更好的稳定性及精确性；RAB具有更好的灵活性，有助于在复杂弯曲的支气管树中到达更外周的病变；此外，Monarch^®^系统提供连续的可视性，而Ion^®^系统的光纤形状传感技术提供对于支气管镜形状与位置的实时感知与跟踪。

RAB也存在一些局限性：（1）RAB缺乏触觉反馈，增加了操作时出血、气道损伤等严重并发症的风险；（2）虽然RAB的灵活性和可视性优于传统支气管镜，但是由于远端支气管和气道压力等原因，一定程度上限制了Monarch^®^系统对亚段支气管进行深入活检取样；（3）Monarch^®^系统定位依靠电磁导航装置，可能会被起搏器等电磁装置以及金属物干扰；（4）Ion^®^系统定位取决于镜身鞘管中的光纤传感装置，在穿刺活检过程中缺乏实时视觉反馈，同时存在CTBD，为了确认活检工具和目标结节的位置关系，术中须使用影像辅助技术，未来这些定位技术与RAB系统的整合值得期待；（5）RAB仍处于起步阶段，成本较高、手术时间较长以及对手术室要求较高。

## 6 展望

基于Ion^®^系统和Monarch^®^系统的前瞻性、观察性队列研究（NCT04740047和NCT04182815—TARGET：30个中心1200例）正在积极招募，主要终点为手术相关并发症，次要终点包括术后随访24个月的最终诊断率，拟于2023年12月完成^[50]^，评估Ion^®^系统在I期非小细胞肺癌或肺转移瘤术中辅助定位（NCT04987281）可行性的II期临床REPLACING研究也正在招募中。2022年3月30日，四川大学华西医院成功完成全国首例国产RAB微创^®^系统肺结节活检。尽管RAB在周围型肺结节活检与PTNB具有相似的诊断率和更低的并发症发生率，但是RAB仍需要朝着设计研发可弯曲且可靠的支气管镜、提高机器人系统的集成度、实现位置与视觉双重导航、降低位置偏移、活检充分确切的方向完善。
